# RRS1 silencing suppresses colorectal cancer cell proliferation and tumorigenesis by inhibiting G2/M progression and angiogenesis

**DOI:** 10.18632/oncotarget.20897

**Published:** 2017-09-15

**Authors:** Xin-Lin Wu, Zhi-Wen Yang, Li He, Pei-De Dong, Ming-Xing Hou, Xing-Kai Meng, Hai-Ping Zhao, Zhao-Yang Wang, Feng Wang, Baoluri, Wurenqimuge, Agudamu, Yong-Feng Jia, Lin Shi

**Affiliations:** ^1^ Department of Gastrointestinal Surgery, The Affiliated Hospital of Inner Mongolia Medical University, Hohhot 010059, Inner Mongolian Autonomous Region, China; ^2^ Department of Hepatobiliary Surgery, The Affiliated Hospital of Inner Mongolia Medical University, Hohhot 010059, Inner Mongolian Autonomous Region, China; ^3^ Department of Pathology, The Affiliated Hospital of Inner Mongolia Medical University, Hohhot 010059, Inner Mongolian Autonomous Region, China; ^4^ Institute of Pathology and Pathophysiology, Inner Mongolia Medical University, Hohhot 010059, Inner Mongolian Autonomous Region, China

**Keywords:** colorectal cancer, RRS1, angiogenesis, cell cycle arrest, apoptosis

## Abstract

Colorectal cancer (CRC) is one of the most common malignancies worldwide. Ribosome biogenesis regulatory protein homolog (RRS1) is an essential factor involved in ribosome biogenesis, while its role in CRC remains largely unclear. Here, we found that RRS1 expression was significantly higher in CRC tissues compared with adjacent normal tissues. RRS1 High expression also predicted poor overall survival of CRC patients. Knockdown of RRS1 induced the G2/M cell cycle arrest, apoptosis and suppressed the proliferation of RKO and HCT-116 CRC cells. Furthermore, angiogenesis was also reduced in CRC cells after RRS1 knockdown. In addition, suppression of RRS1 blunted the tumor formation of CRC cells in nude mice. At the molecular level, silencing of RRS1 decreased the expression of M-phase inducer phosphatase 3 (CDC25C), Cyclin-dependent kinase 1 (CDK1), antigen KI-67 (KI67) and increased the protein level of cyclin-dependent kinase inhibitor 1 (CDKN1A) and tumor suppressor p53 (p53). Taken together, our findings provide evidence that RRS1 may promote the development of colon cancer. Therefore, targeting RRS1 may be a promising therapeutic strategy for CRC patients.

## INTRODUCTION

Colorectal cancer (CRC) is the third most common malignancy and fourth leading cause of cancer death worldwide [1, 2]. Once diagnosed at the early stage, surgery is the prior treatment for CRC patients. As expected, most cases relapse and develop into the advanced stage and adjuvant chemotherapy is performed to alleviate tumor progression and metastasis [3, 4]. However, the outcomes have not yielded expected success.

Genomic alterations are essential risk factors that trigger the initiation and progression of CRC [5, 6]. Among these, KRAS, APC and p53 are the most frequently mutated genes found in CRC specimens [7–10]. Indeed, functional studies *in vitro* and *in vivo* demonstrate their critical role in CRC development [10, 11]. Based on these studies, targeted therapies against the proto-oncogenes or tumor suppressors have evolved, while the effectiveness is very limited [12–14]. Therefore, there is a constant need to explore novel drug targets that are involved in CRC progression.

Increased protein synthesis, which is accompanied with enhanced ribosome biogenesis, is a primary feature of cancer cell proliferation, including CRC. Ribosome biogenesis regulatory protein homolog (RRS1) is a conserved protein in eukaryotes and related studies start in saccharomyces cerevisiae [15]. Together with Rpf2, it promotes the maturation of 60S ribosome subunit [16]. It is required for cell cycle transition by balancing with other ribosome components [17]. Dys-regulation of RRS1 is involved endoplasmic reticulum stress response of Huntington disease [18]. Despite extensive studies into its fundamental, the role of RRS1 in colorectal cancer remains largely unclear.

In this study, we identified RRS1 as a novel proto-oncogene in CRC. Increased RRS1 level was found in CRC specimens and negatively correlated with survival rate. Knockdown of RRS1 in CRC cells RKO and HCT116 induced apoptosis and suppressed G2/M cell cycle transition, angiogenesis, cell proliferation and xenografted tumor formation. Mechanistically, cell cycle related factors and p53 pathway are major downstream targets.

## RESULTS

### RRS1 is a potential biomarker for CRC patients

**Figure 1:**
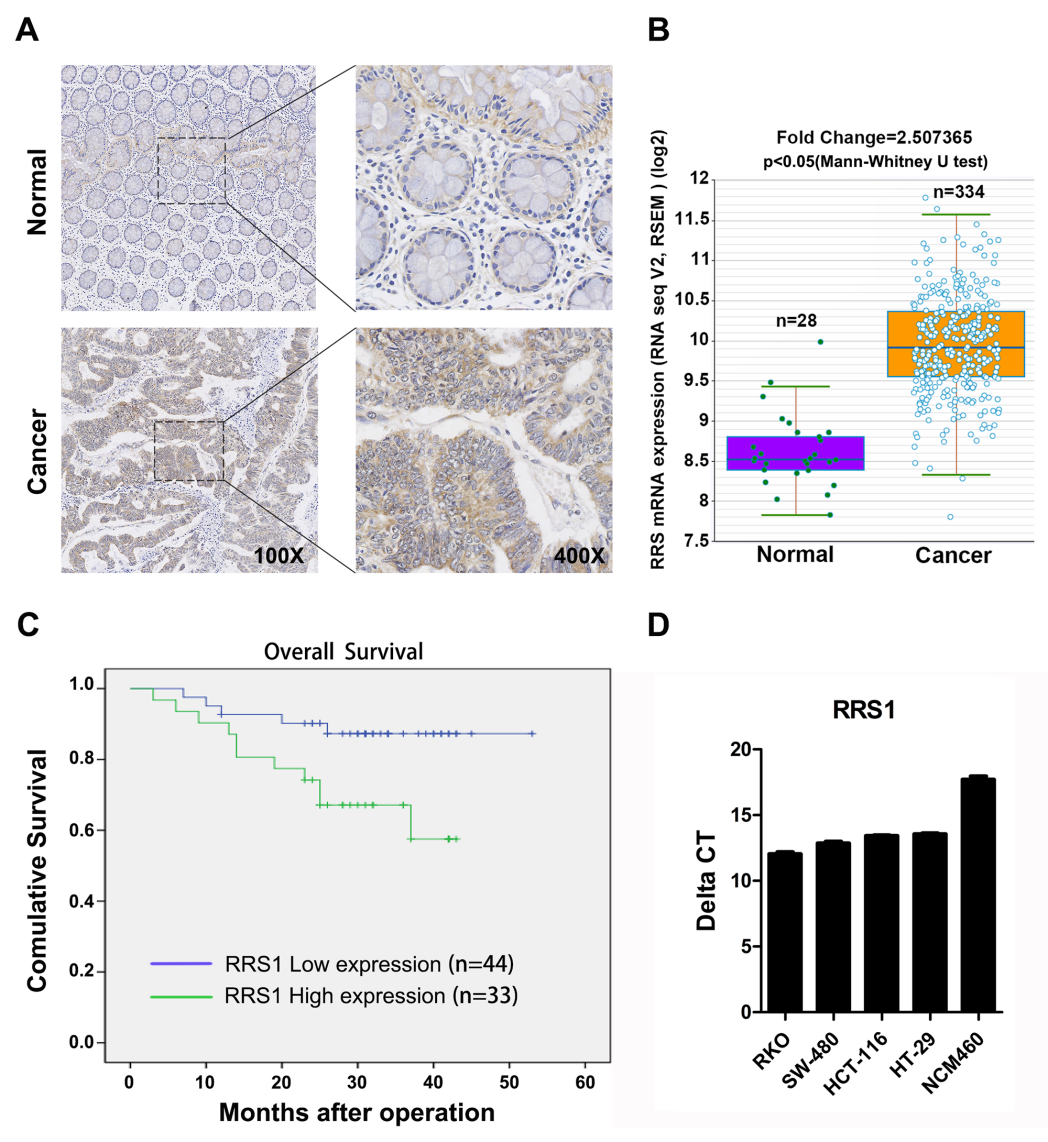
RRS1 is overexpressed in human colon cancer tissue and cells **(A)** Immunohistochemical (IHC) staining analysis of RRS1 protein expression in primary colon cancer tissues and adjacent noncancerous tissues. **(B)** The expression levels of RRS1 in colon cancer and normal tissues of the patients from the Cancer Genome Atlas (TCGA) database. **(C)** Overall survival (OS) curve of 77 primary colon tumors. The survival rate of RRS1-high expression group (n=33) is significantly lower than that of the RRS1-low expression group (n=44). **(D)** Expression of RRS1 was measured by q-PCR in four human colon cancer cell lines (RKO, SW-480, HCT-116, HT-29) and one normal colorectal cell line (NCM460). The relative expression of RRS1 is shown as delta CT (CT^RRS1^-CT^GAPDH^).

To explore the clinical relevance of our study, 77 CRC tissues and 16 paired adjacent normal tissues were collected and RRS1 expression was determined. Based on immunohistochemistry assay, we found that RRS1 expression was significantly higher in the tumor areas (Figure 1A, Table 1). The clinicopathological characteristics revealed that RRS1 expression was relatively increased in higher T or N stage (Table 2). Next, we analyzed RRS1 expression in 334 colon tumor tissue and 28 normal tissue using RNA sequencing. The results showed that RRS1 was significantly over-expressed in the colon tumor tissues (Figure 1B). Moreover, a total of 77 patients was divided into RRS1 low expression group (n=33) and RRS1 high expression group (n=44). The survival of CRC patients with low RRS1 expression was better than those who had high RRS1 level (Figure 1C). We also found that the RRS1 mRNA level was highly expressed in four CRC cell lines compared with normal colorectal cells (Figure 1D). Taken together, RRS1 is a useful biomarker in CRC patients that can be used to monitor the tumor progression after operation.

**Table 1:** IHC staining analysis for RRS1 between cancer and normal tissue (Mann-Whitney U test)

Variables	RRS1		Total no.	P value
	Low expression, no.	High expression, no.		
**Cancer**	44	33	77	
**Normal**	16	0	16	0.001
**Total**	60	33	93	

**Table 2:** Association among clinicopathological variables and RRS1 expression

Variables	RRS1		Total no.	P value
	Low expression, no.(%)	High expression, no.(%)		
**Tumor volume**				
**≤5cm**	**28(50.9)**	**27(49.1)**	**55**	**0.083**
**>5cm**	**16(72.7)**	**6(27.3)**	**22**	
**Gender**				
**Male**	**33(67.3)**	**16(32.7)**	**49**	**0.017**
**Female**	**11(39.3)**	**17(60.7)**	**28**	
**Age**				
**≤59**	**26(61.9)**	**16(38.1)**	**42**	**0.358**
**>59**	**18(51.4)**	**17(48.6)**	**35**	
**T Stage**				
**II/III**	**34(69.4)**	**15(30.6)**	**49**	**0.004**
**IV**	**10(35.7)**	**18(64.3)**	**28**	
**N Stage**				
**N0**	**21(77.8)**	**6(22.2)**	**27**	**0.008**
**N1/2**	**23(46.0)**	**27(54.0)**	**50**	

### RRS1 silencing suppresses CRC cell proliferation

**Figure 2:**
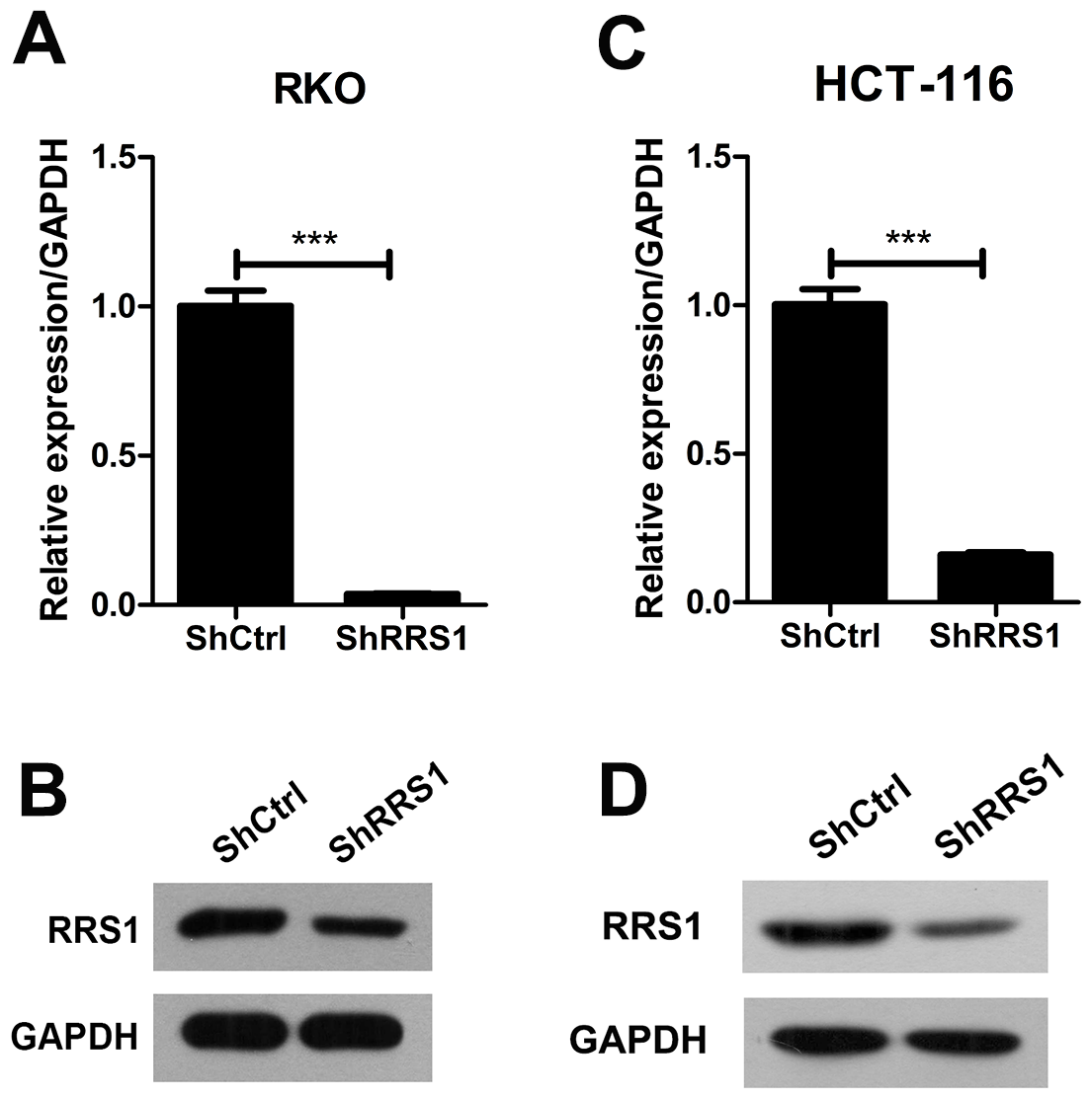
RRS1 was efficiently knockdown in human colon cancer cells **(A-B)** Quantitative RT-PCR and Western blot analysis revealed the RRS1 expression was efficiently knockdown in the RKO cells. **(C-D)** Quantitative RT-PCR and Western blot analysis revealed the RRS1 expression was efficiently knockdown in the HCT116 cells. ***P<0.001.

**Figure 3:**
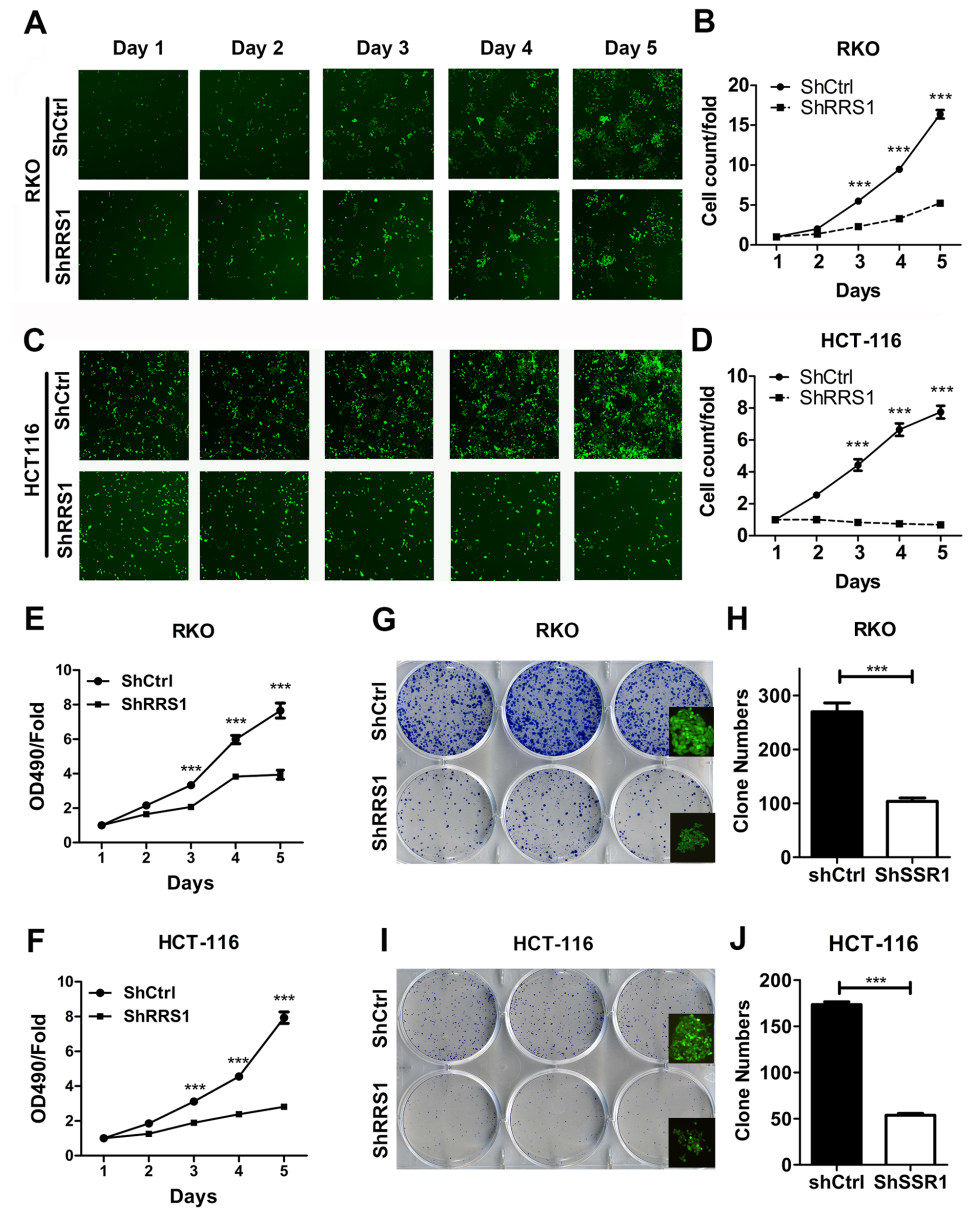
Knockdown of RRS1 inhibited human colon cancer cells proliferation **(A)** Representative pictures of RKO cells infected with shCtrl (top) and shRRS1 (bottom) via multiparametric high-content screening (HCS) every day for five days. **(B)** Statistics for HCS assay in the RKO cells. **(C)** Representative pictures of HCT116 cells infected with shCtrl (top) and shRRS1 (bottom) via multiparametric high-content screening (HCS) every day for five days. **(D)** Statistics for HCS assay in the HCT116 cells. **(E-F)** RRS1 knockdown inhibits the RKO cells (E) and HCT116 cell (F) proliferation that was determined by MTT assay. **(G-H)** Colony formation analysis of RKO cells that were infected with shCtrl or shRRS1 lentivirus. **(I-J)** Colony formation analysis of HCT116 cells that were infected with shCtrl or shRRS1 lentivirus. ***P<0.001.

RRS1 is up-regulated in CRC specimens, whether it is critical for CRC progression remains unknown. To address this question, we knocked down RRS1 in two CRC cell lines RKO and HCT116. qRT-PCR and western blot results showed that RRS1 was silenced in both cells (Figure 2). Cell culture plates were scanned and cells were counted from day 1 to day 5. Cell proliferation rate was largely inhibited from day 3 in RKO cells after RRS1 knockdown (Figure 3A-3B and 3E). Interestingly, HCT116 cell viability was almost completely blunted after RRS1 knockdown (Figure 3C-3D and 3F). These results indicated that RRS1 was critical for CRC cells survival. In line with this, 2 to 3-fold more colonies were formed in shCtrl RKO cells compared with shRRS1 RKO cells (Figure 3G and 3H). Furthermore, approximately 70% reduction of colony numbers was observed in shRRS1 HCT116 cells compared to control cells (Figure 3I and 3J). In conclusion, targeting RRS1 suppresses CRC cells survival.

### Reduction of RRS1 blunts tumorigenic capacity of CRC cells

**Figure 4:**
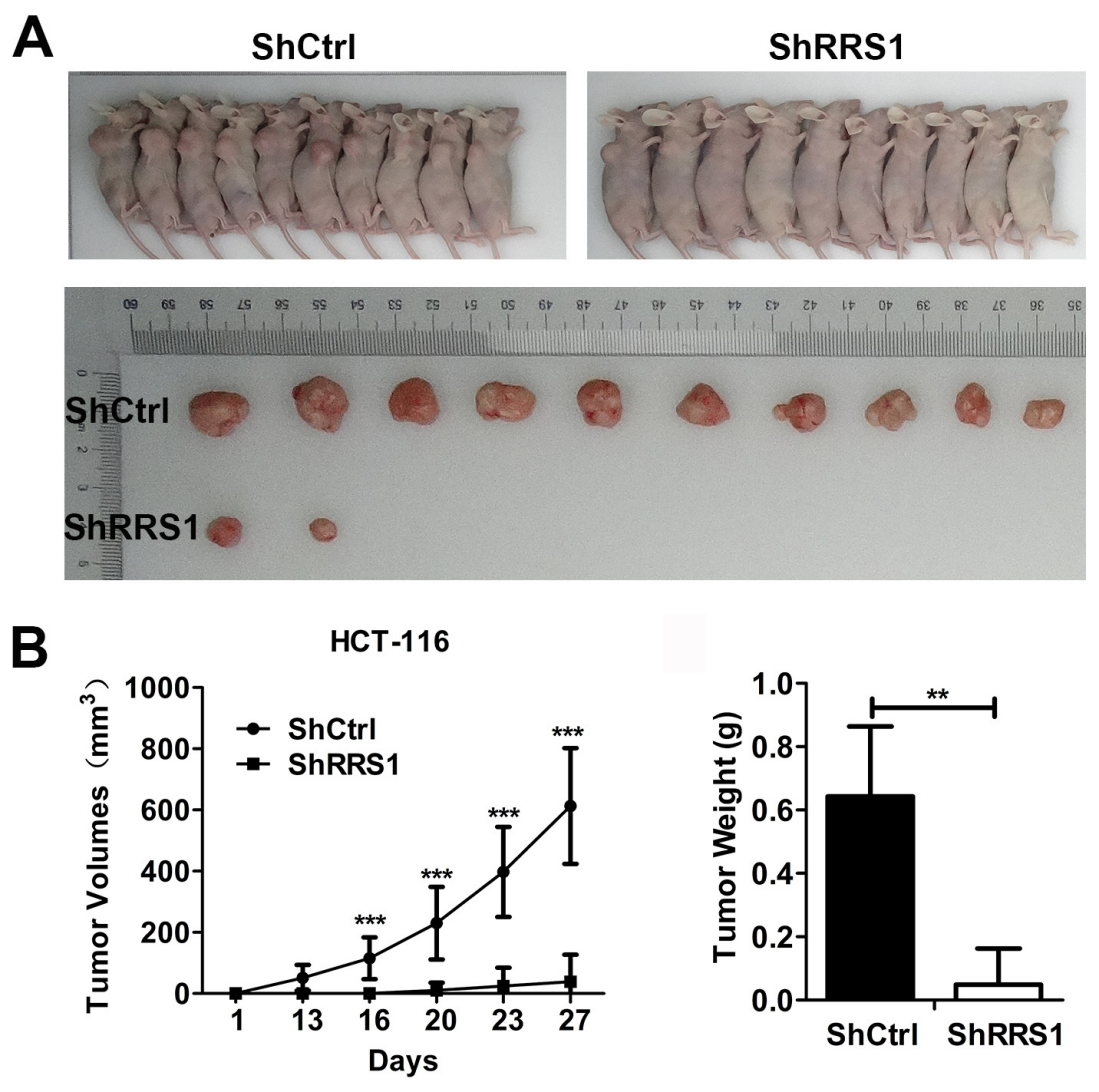
Knockdown of RRS1 represses tumor formation of CRC cells *in vivo* **(A)** Subcutaneous tumors in nude mice and isolated tumors after 4 weeks formed by HCT116 cells infected with shCtrl and shRRS1 lentivirus (n=10 in each group). **(B)** Tumor growth curve of xenografts in nude mice (n=10 in each group). Xenograft volumes were calculated using the formula v=0.5ab^2^ (a: long diameter, b: short diameter). ***P<0.001. **(C)** Statistics for the tumor weight of xenografts (n=10 in each group). **P<0.01.

To explore the role of RRS1 in the tumorigenesis of CRC cells, we transplanted CRC cells expressing shCtrl or shRRS1 into nude mice. We found that all of the ten nude mice transplanted with cells expressing shCtrl exhibited visible and large tumors, while cells expressing shRRS1 only developed two smaller tumors in ten nude mice (Figure 4A). Tumor volume was evaluated from day 1 to day 27 and the results showed that tumorigenesis of CRC was dramatically blunted after RRS1 silencing (Figure 4B and 4C). These findings indicated that RRS1 was essential for CRC cells tumorigenesis *in vivo*.

### Knockdown of RRS1 induces G2/M cell cycle arrest, apoptosis and inhibits angiogenesis

**Figure 5:**
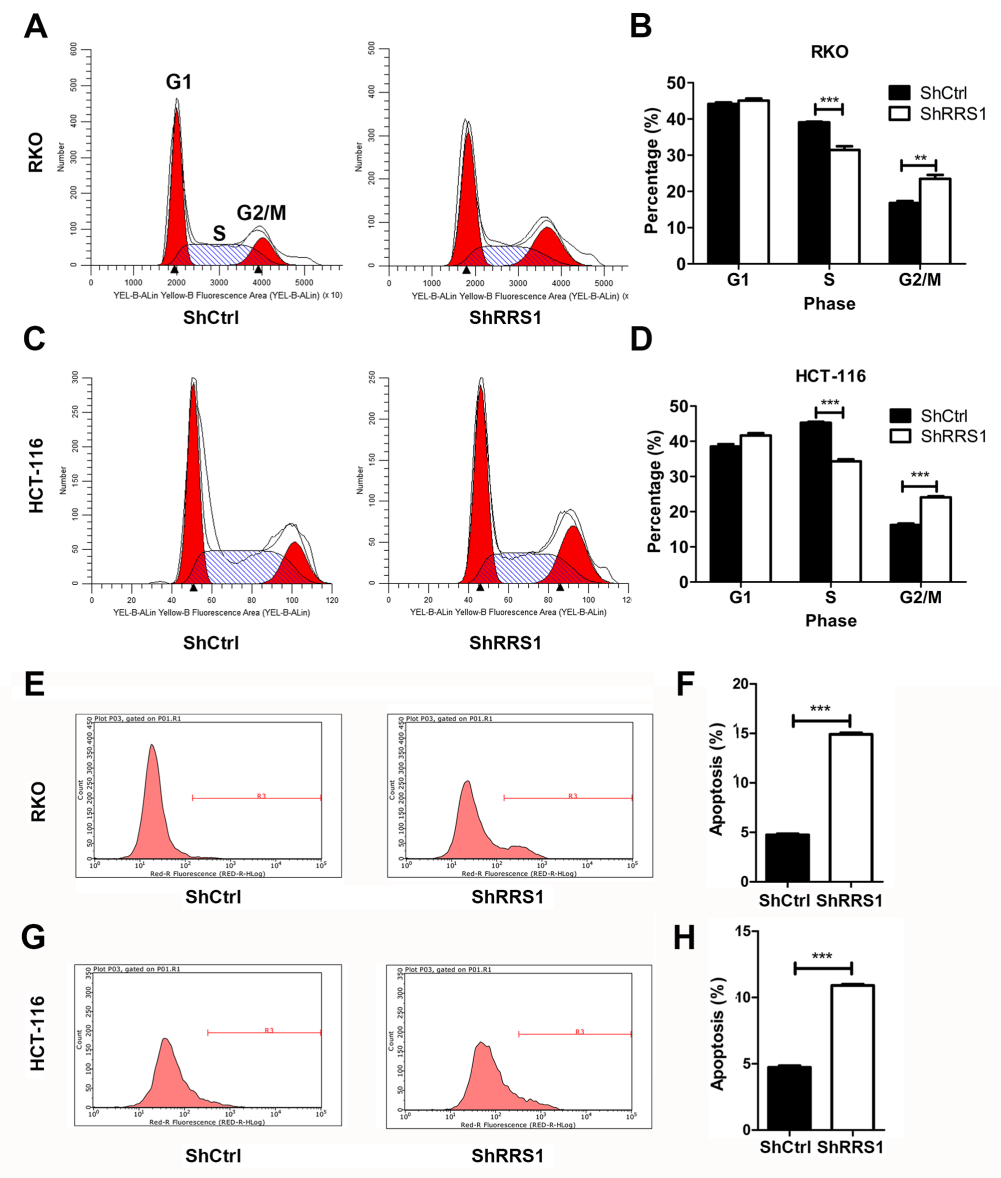
Knockdown of RRS1 induces cell cycle G2/M arrest and apoptosis **(A-B)** Flow cytometry analysis of cell cycle revealed that RRS1 knockdown induced RKO cell cycle arrested at G2/M phase. **(C-D)** Flow cytometry analysis of cell cycle revealed that RRS1 knockdown induced HCT-116 cell cycle arrested at G2/M phase. **(E-F)** Flow cytometry analysis of apoptosis revealed that RRS1 knockdown induced RKO cell apoptosis. **(G-H)** Flow cytometry analysis of apoptosis revealed that RRS1 knockdown induced HCT-116 cell apoptosis. **P<0.01, ***P<0.001.

Accelerated cell cycle progression is a common feature of tumor cells with activated proliferation rate. Next, we analyzed the cell cycle division of CRC cells expressing shCtrl or shRRS1. Minimal difference of G1 phase was found between shCtrl and shRRS1 RKO or HCT116 cells (Figure 5A-5D). The percent of shRRS1 cells in S phase was lower compared with that of shCtrl cells (Figure 5A-5D). In addition, we observed that there was a higher percentage of RKO and HCT116 cells expressing shRRS1 in G2/M phase compared with shCtrl cells (Figure 5A-5D), indicating that RRS1 silencing results in cell cycle arrest of CRC cells in G2/M phase. We also analyzed the effect of RRS1 knockdown on apoptosis of CRC cells. We found that RRS1 silencing significantly induced the apoptosis of CRC cells (Figure 5E-5H).

**Figure 6:**
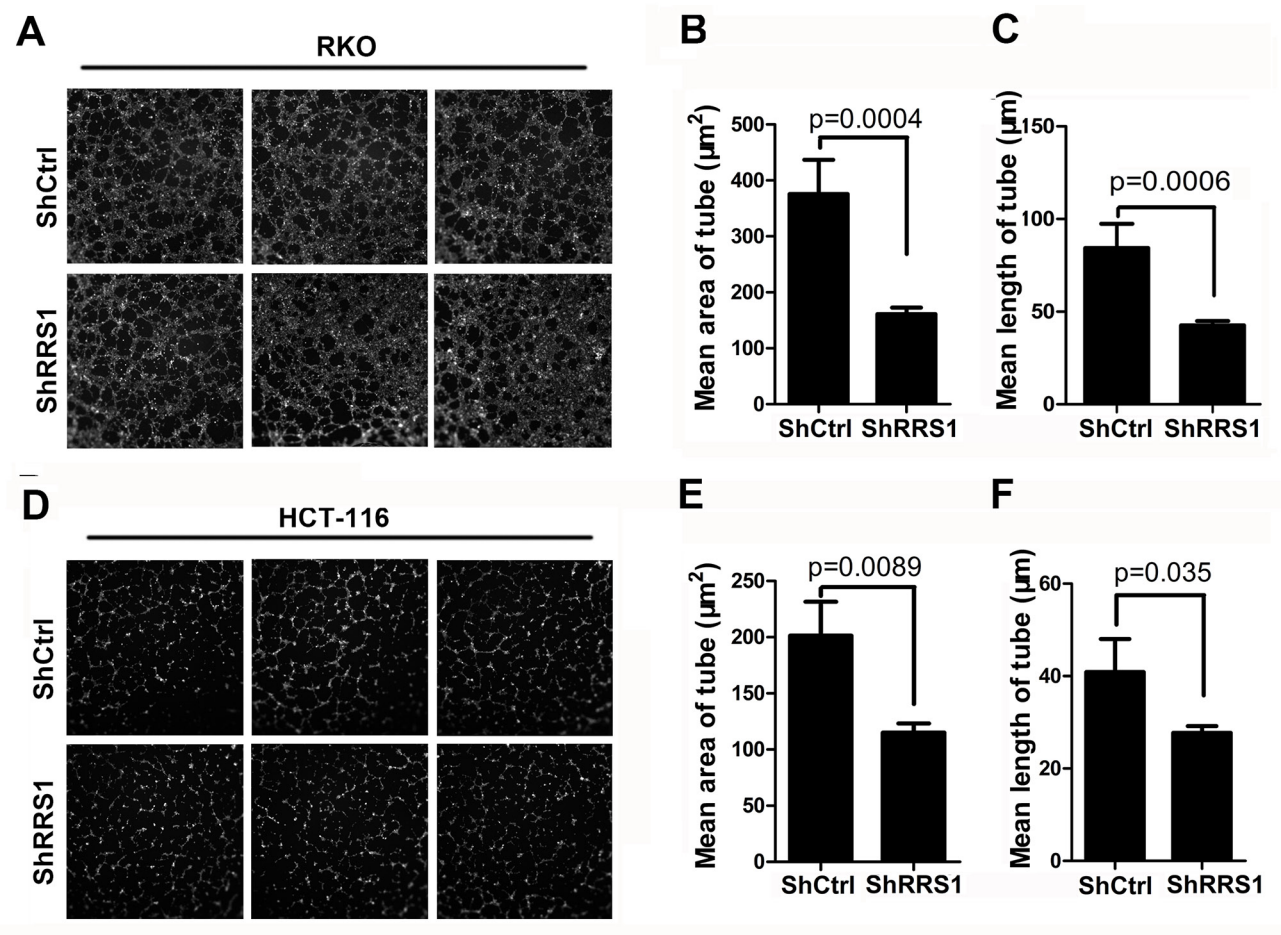
Knockdown of RRS1 represses colon cancer cell angiogenesis **(A)** Images of endothelial cell tube formation assay in shCtrl or shRRS1 RKO cells. **(B-C)** Mean area (B) and length (C) of tube analysis of endothelial cell tube formation assay described in (A). **(D)** Images of endothelial cell tube formation assay in shCtrl or shRRS1 HCT-116 cells. **(E-F)** Mean area (B) and length (C) of tube analysis of endothelial cell tube formation assay described in (D).

Enhanced angiogenesis provides energy fuel that promotes tumor cell proliferation and growth. In this study, we revealed that angiogenesis was suppressed in RKO cells (Figure 6A-6C) and HCT116 cells (Figure 6D-6F) after RRS1 knockdown. Taken together, silencing of RRS1 blunts cell proliferation and tumorigenesis of CRC cells at least partly through promoting G2/M cell cycle arrest, apoptosis and inhibiting blood vessel formation.

### p53 signaling pathway and cell cycle related cytokines locate downstream of RRS1

**Figure 7:**
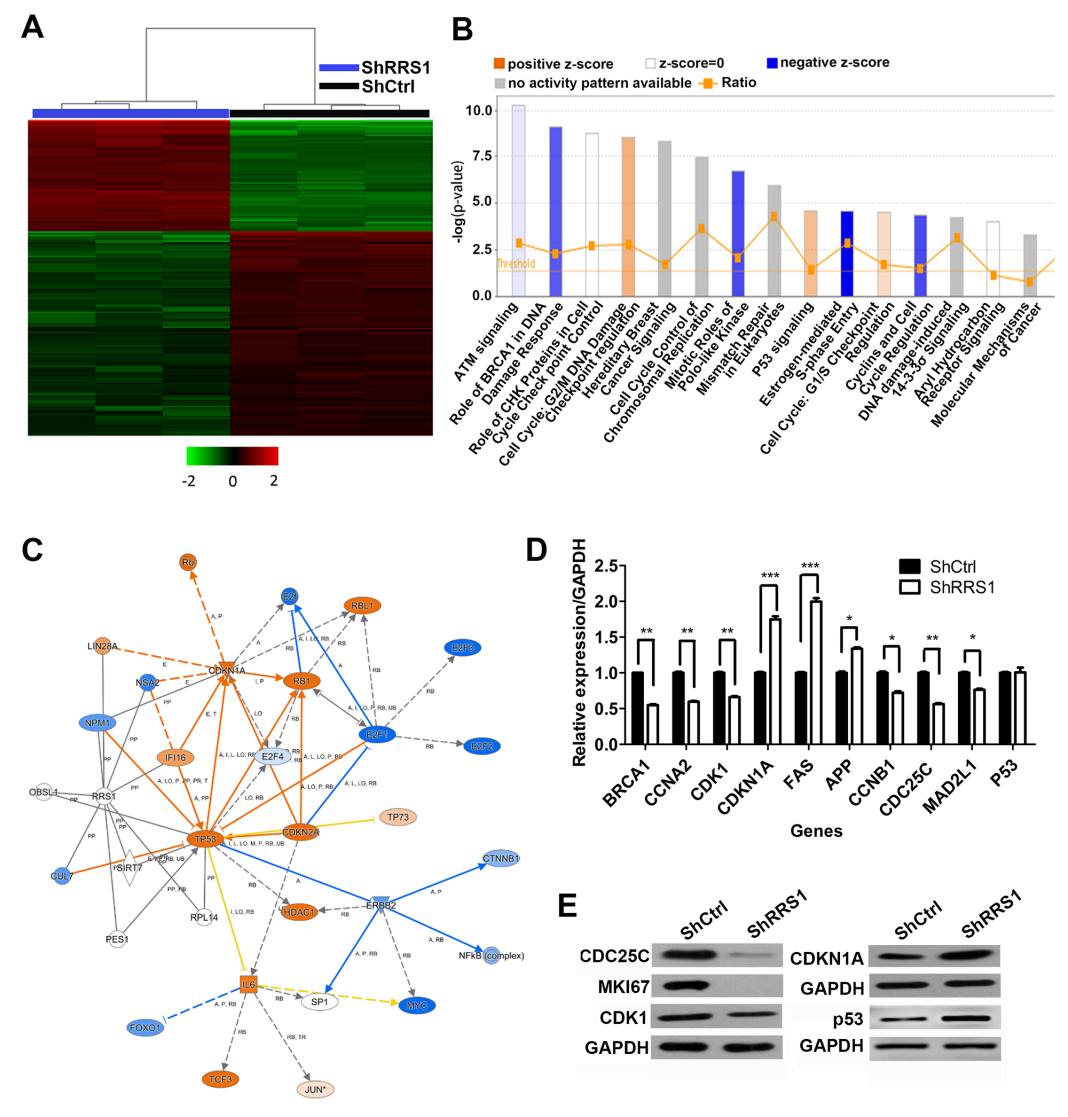
Disruption of multiple key pathways are involved in colon cancer cells after RRS1 knockdown **(A)** Heat map representation of 905 genes (324 genes up-regulated and 581 genes down-regulated) showed significant differential expression patterns in HCT116 cells infected with lentivirus expressing shCtrl and shRRS1 (criteria P<0.05, absolute fold change >1.5). **(B)** Functional pathway enrichment of differential genes was analyzed using IPA software. **(C)** Interactional network was constructed between genes involved in p53 pathway. Blue circles represent down-regulated genes, red circles represent up-regulated and genes of gray circles represent no expression changing. **(D)** The expression of indicated downstream targets of p53 signaling. **(E)** The expression of MKI67, CDC25C, CDK1, CDKN1A and p53 that was examined by Western blot assay. GAPDH is used as an internal control.

To understand the molecular mechanisms, global gene expression profile of shCtrl or shRRS1 CRC cells were examined using microarray platform. Totally, 324 genes were up-regulated and 582 genes were down-regulated after RRS1 silencing (fold change>1.5, p<0.05) (Figure 7A). Furthermore, pathway enrichment analysis showed that multiple pathways were enriched, including DNA damage response, cell cycle and p53 pathways (Figure 7B). We focused on p53 pathway because of its importance in multiple type of cancer. Our results showed that the level of many downstream genes of p53 increased or decreased by RRS1 knockdown (Figure 7C). To validate these results, we focused on genes related both with p53 pathway and the cell cycle and found that BRCA1, CDK1, CCNB1, CDC25C, CCNA2 and MAD2L1 were down-regulated, while CDKN1A, FAS and APP were up-regulated (Figure 7D). Consistently, at the protein level, decreased CDC25C, CDK1, MKI67 and enhanced CDKN1A expression was found in RRS1 silencing CRC cells (Figure 7E). Interestingly, the mRNA level of p53 remained unchanged while the protein level of p53 increased in shRRS1 cells, indicating that RRS1 decreased the protein stability of p53 (Figure 7D and 7E). These results indicated that RRS1 knockdown might inhibit the cell proliferation mainly by blocking cell cycle progression via activating p53 pathway.

## DISCUSSION

Because of limited therapeutic options for CRC patients, understanding the molecular events that are critical for CRC progression is urgent. In this study, we reported for the first time that RRS1 was a pathogenic protein in CRC. Patients with higher RRS1 expression had a worse overall survival compared with those with lower expression of RRS1. Silencing of RRS1 inhibited the proliferation and tumorigenesis of CRC cells in nude mice. These results revealed that targeting RRS1 might be a promising strategy for CRC patients.

The primary function of RRS1 is mainly involved in ribosome biogenesis, which is essential for protein synthesis. Enhanced cancer cell proliferation is always accompanied with increased protein synthesis [19]. Recent studies confirmed a definite role of some ribosome proteins in cancer development [20]. Here in this study, we explored the role of RRS1 in CRC development. We found that knockdown of RRS1 significantly blunted the proliferation rate and tumorigenesis ability of CRC cells. It has been reported that RRS1 mutation delayed G1 to S phase transition, suggesting that RRS1 is essential for cell cycle transition [17]. Our results revealed that reduction of RRS1 inhibited cell cycle transition from G2 to M phase. Moreover, the expression of CCNA2, CDK1, CCNB1, CDC25C and MAD2L1 was decreased and that of CDKN1A, APP was increased. Cell proliferation marker KI67 was also down-regulated after RRS1 knockdown. A previous study found that dys-regulated ribosome biogenesis sensitized cells to death via p53 activation [21]. Importantly, we found that RRS1 knockdown up-regulated p53 protein level but not the mRNA level, indicating that RRS1 down-regulates p53 in a post-transcriptional dependent manner. These results suggested that RRS1 silencing promoted cell cycle arrest partly through up-regulation or down-regulation of cell cycle related proteins. Cancer cells had a characteristic of suppressed apoptosis. Here we found that RRS1 knockdown induced apoptosis of CRC cells. The enhanced cell cycle arrest and apoptosis might explain why the proliferation of CRC cells was strikingly suppressed by RRS1 knockdown.

Increased angiogenesis is one of the hallmarks of cancer and is positively correlated with cancer metastasis [22, 23]. Importantly, high metastatic rate is the major cause of death in CRC patients [24]. Thus, targeting angiogenesis might be an effective way to treat advanced CRC. Our results showed that RRS1 silencing suppressed the angiogenesis of RKO cells. These findings indicated that RRS1 might be a promising target for metastatic CRC patients.

In summary, our study provided the first evidence that RRS1 was a novel oncogene for CRC. Silencing of RRS1 suppressed cell proliferation and tumor formation of CRC cells, mainly through cell cycle arrest at the G2/M phase, enhanced apoptosis and angiogenesis inhibition. At the molecular level, cell cycle transition factors were involved in the pathogenic effect of RRS1 in CRC cells.

## MATERIALS AND METHODS

### Patient information and TCGA colon cancer mRNA database and analysis

All 77 patients with colon cancer and 16 adjacent normal tissues which were taken from the area more than 10cm away from primary neoplasms were enrolled from 2012 to 2014. The median age of patients was 59 years (range: 49-68) at the time of surgery, and the median follow-up time was 31 months post-operatively (range: 25-37 months). The study was approved by the Ethics Committee of The Affiliated Hospital of Inner Mongolia Medical University. A written informed consent was obtained from all patients. Transcriptome expression datasets and the corresponding clinical information were downloaded from websites of The Cancer Genome Atlas (http://cancergenome.nih.gov). Total 362 samples, which contain transcriptional expression data of 334 tumor tissues and 28 normal tissues, were available for this analysis.

### Cell culture

Human colorectal cancer cell lines RKO and HCT116 were cultured in 1640 medium (Invitrogen), supplemented with 10% fetal bovine serum (Corning) and 1% penicillin and streptomycin solution (Corning). All the cells were cultured at 37°C with 5% CO_2_.

### Total RNA isolation and quantitative real-time PCR

Total RNA was isolated from indicated cells using Trizol reagent (Invitrogen) and Ultrapure RNA Kit from CWBIO (Beijing, China) according to the manufacturer’s instructions. 0.8 microgram of total RNA was reverse-transcribed using M-MLV reverse transcriptase (Promega). Quantitative real-time PCR was performed on a real-time PCR machine TP800 (Takara) using SYBR master mixture (Takara). The primer sequences are as follows: RRS1 forward, 5′- CCCTACCGGACACCAGAGTAA-3′, RRS1 reverse, 5′- CCGAAAAGGGGTTGAAACTTCC-3′; and GAPDH forward, 5′- TGACTTCAACAGCGACACCCA-3′, GAPDH reverse, 5′- CACCCTGTTGCTGTAGCCAAA-3. The relative RRS1 expression was normalized to GAPDH, and data analysis was conducted using the comparative CT method.

### Western blot

Total protein was isolated from indicated cells using lysis buffer (Beyotime) and concentration was determined by BCA protein assay kit (Beyotime). Total protein was separated on a 12 % sodium dodecyl sulfate polyacrylamide gel electrophoresis (SDS-PAGE) and transferred to polyvinylidenefluoride membrane (PVDF; Millipore, USA). The membranes were blocked with 5% skim milk for 1 hour at room temperature and immunoblotted with primary antibodies at 4°C overnight. Antibody against CDC25C was from Cell Signaling Technology. Antibody against CDKN1A and MKI67 were from Abcam.

### Packaging of sh-RRS1 lentivirus

The lentivirus system is composed three vectors: pGCSIL-GFP (stably expressed shRNA fused with a GFP marker), pHelper1.0 (gag/pol element) and Helper2.0 (VSVG element). shRNA targeting human RRS1 (5′- GCTGCCTTCATTGAGTTTA -3′) and the control shRNA used as negative control (5′- TTCTCCGAACGTGTCACGT-3′) were designed, synthesized and cloned into the pGCSIL-GFP vector by GeneChem Corporation (Shanghai, China). The three vectors were mixed and transfected to 293T cells with Lipofectamine TM 2000 (Invitrogen, Shanghai, China). After 48h transfection, viral supernatants were collected, centrifuged and filtered through 0.45μm polyvinylidene fluoride membranes. Then the viral supernatants were used to infected RKO and HCT116 cells and the cells were lysed 72 hours after virus infection for real-time PCR and western blot assays.

### Immunohistochemistry

A total of 93 paired Formalin-fixed, paraffin-embedded (FFPE) tissue samples were stained using primary anti-RRS1 antibody (ab188161, Abcam). The intensity of RRS1 expression was graded as follows: negative = score 0, weak = score 1, moderated = score 2 and strong = score 3. Extent of staining was grouped according to the percentage of high-staining cells in the cancer nest: negative = score 0, 1% to 25% = score 1, 26% to 50% = score 2, 51% to 75% = score 3 and 76% to 100% = score 4. The final quantitation of each staining was obtained by multiplying the two scores. Low expression means refers to a RRS1 score ing≤6, and high expression means refers to a RRS1 scoring score >6. Immuno-reactivity was assessed independently by two expert pathologists blind to all clinical data.

### Colony formation assay

Cells infected with shRNA lentivirus targeting negative control or RRS1 were seeded in six-well plates (800 cells/well). After cultured at 37°C for 14 days, colonies were formed. Then the cells were fixed with methanol for 30 min and stained with Giemsa solution for 10 min. The number of colonies (> 50 cells/colony) was quantified using a fluorescence microscopy (Olympus).

### Tube formation assay

Briefly, Matrigel Basement Membrane Matrix (70μl for each well, Corning) were pipetted into each well of a 96-well plate and polymerized for 30 minutes at 37°C. Total 2×10^4^ HUVECs were suspended in 100μl conditioned medium from RRS1 knockdown group or NC group and incubated for 8 hours at 37°C, 5% CO_2_. The measurement is AngiogenicIndexCh1 which is defined as 1000 × Total Area of Connected Tubes/Total Image Area was determined by the ArrayScan™ HCS software (Cellomics Inc).

### Cell cycle assay

Cell cycle progression was examined on a flow cytometer using propidium iodide (PI) staining. Cells infected with shRNA lentivirus against NC or RRS1 were seeded in six-well culture plates and cultured to 80% confluence. Cell cycle was analyzed by PI staining of nuclei. PI absorbance was determined by fluorescence activated cell sorting on a flow cytometry (FACSCalibur, Becton Dickinson).

### High-content screening for cell proliferation assay

Cell viability was measured via multiparametric high-content screening (HCS). shCtrl or shRRS1 RKO and HCT116 cells were cultured in 96-well plates for 5 days. Cell proliferation of each wells was determined by the ArrayScan™ HCS software (Cellomics Inc). The fluorescence-imaging microscope could automatically identify stained cells and analyzed the intensity and distribution of fluorescence. Images were acquired using appropriate filters by 20 × objective and stored in a Microsoft SQL database.

### MTT for cell proliferation assay

RKO cells and HCT-116 cells infected with NC lentivirus or RRS1-shRNA lentivirus were seeded in 96-well plates at a density of 2000 cells/well and incubated at 37°C for 1, 2, 3, 4, and 5 days, respectively. After washed by PBS two times, each well was added into 3-(4,5-dimethyl-2-yl)-2,5-diphenyltetrazolium bromide (MTT) solution (5 mg/mL). After 4 hours of incubation, supernatants in each well were removed and then 100 μL of dimethyl sulfoxide (DMSO) was added to solubilize the formazan salt. Ten minutes later, the optical density (OD) was measured at 490 nm by using a micro-plate reader.

### Apoptosis assay

Cell apoptosis was determined by annexin V-APC apoptosis detection kit (Ebioscience, USA) following the manufacturer’s protocol. Indicated cells were washed with PBS, and resuspended with staining buffer. 5μl annexin V-APC was added into a total of 100 μl cell suspension. The mix was incubated for 15 min at room temperature, and then subjected to flow cytometry analysis (FACSCalibur, Becton-Dickinson, USA).

### *In vivo* xenograft assay

A total of 4*10^6^ HCT116 cells were subcutaneously injected into the right armpit of the 4-week old male mice. Slide caliper rule was used to measure the xenograft diameters every other day until day 28. The xenograft tumor volume was calculated with the following formula: v=0.5ab^2^ (a=long diameter of the tumor, b=short diameter of the tumor, and v=volume).

### Microarray

Total RNA from HCT116 cells infected with NC-lentivirus (n=3) or shRRS1-lentivirus (n=3) was extracted using Trizol reagents. The quantity and quality of extracted RNA were detected by NanoDrop 2000 and Agilent Bioanalyzer 2100. Affymetrix human GeneChipprimeview was used for microarray processing to determine gene expression profile depending on the manufacturer’s instruction. Significantly different genes between HCT116 cell treated with shCtrl and shRRS1 were identified the follow criteria: P<0.05 and the absolute fold change >1.5. The biofunction and pathway enrichment analysis were performed using IPA®Software (http://www.ingenuity.com).

### Statistical analysis

The statistical analyses were performed using SPSS version 16.0 (SPSS, Inc., Chicago, IL). The raw data were presented as the mean ± standard error of mean (SEM) of at least three independent repeats if no any other statements. Students’ t tests were applied to analyze the differences between two groups. Differences among groups were determined by one-way or two-way analysis of variance (ANOVA) with repeated measures, followed by the Bonferroni post hoc test. Mann-Whitney U test was used for the IHC staining and clinical character analysis. P value of less than 0.05 was considered significant.
